# Propranolol Effective in Suppressing Paroxysmal Sympathetic Hyperactivity Attacks Occurring in the Acute Phase of Diffuse Axonal Injury: A Case Report

**DOI:** 10.7759/cureus.77653

**Published:** 2025-01-19

**Authors:** Satoshi Shinoda, Takafumi Tanei, Hirotaka Nakanishi, Ryuta Saito

**Affiliations:** 1 Neurosurgery, Ogaki Municipal Hospital, Gifu, JPN; 2 Neurosurgery, Nagoya University Graduate School of Medicine, Nagoya, JPN; 3 Neurology, Yokkaichi Municipal Hospital, Mie, JPN

**Keywords:** diffuse axonal injury, paroxysmal sympathetic hyperactivity, post traumatic brain injury, propranolol, β-adrenergic blocker

## Abstract

Paroxysmal sympathetic hyperactivity occurs primarily after severe traumatic brain injury and is characterized by abnormal paroxysmal surges in sympathetic nervous system activity. The clinical symptoms of paroxysmal sympathetic hyperactivity may resemble those of epilepsy or sepsis, leading to delayed diagnosis and inadequate treatment. A 16-year-old male individual suffered a severe brain injury following a traffic accident. His Glasgow Coma Scale score was 4/15 (E1V1M2). Magnetic resonance imaging revealed cerebral contusion in bilateral frontal lobes, genu, and splenium of the corpus callosum, and diffuse axonal injury was diagnosed. Pharmacological therapy with medication to reduce cerebral edema, analgesics, sedatives, and antiepileptic drugs was initiated. On the 7th day, the sedatives were temporarily discontinued, the patient's limbs were held in an extended position with muscle contractions, and also exhibited hyperthermia, tachycardia, sweating, and hypertension, which lasted approximately 30 minutes and occurred multiple times a day. Blood, sputum, and urine cultures were negative, and two electroencephalograms did not detect epileptic discharges, so sepsis and epilepsy were excluded. Baclofen and dantrolene slightly improved muscle stiffness, but the continuous intravenous infusion of sedatives could not be discontinued for more than 40 days because it caused a recurrence of attacks. Propranolol 30 mg/day was added, and paroxysmal sympathetic hyperactivity attacks disappeared. Therefore, sedatives could be discontinued and the patient was transferred to a rehabilitation hospital. Propranolol, an oral β-adrenergic blocker, is one of the effective treatments for paroxysmal sympathetic hyperactivity.

## Introduction

Paroxysmal sympathetic hyperactivity (PSH) is a clinical condition characterized by abnormal paroxysmal surges in sympathetic nervous system activity. Approximately 80% of cases of PSH occur after traumatic brain injury (TBI), but can also occur after hypoxic encephalopathy or stroke [1-3]. The clinical characteristics of PSH include sudden increases in heart rate, respiratory rate, blood pressure, body temperature, diaphoresis, and extended posturing, and because these symptoms appear suddenly, it is called a "PSH attack" [4,5]. Several hypotheses of the pathophysiology of PSH have been proposed, but the excitatory/inhibitory ratio theory is currently accepted [6,7]. Although PSH attacks occur relatively frequently after severe brain injury, they are poorly recognized in clinical practice due to the lack of a clear definition or consistent terminology [1]. Because the clinical symptoms of PSH attacks are similar to those of epileptic seizures or sepsis-related chills, the correct diagnosis of PSH may not be reached. Inconsistency in terminology has led to PSH attacks being named dysautonomia, diencephalic epilepsy, sympathetic storming, or autonomic dysfunction syndrome [8]. In 2014, an expert group defined the syndrome as PSH and established diagnostic criteria and assessment scales [1].

PSH attacks are not caused by epilepsy and therefore cannot be controlled with antiepileptic drugs. Frequent occurrence of PSH attacks prevents patients from getting out of bed and from undergoing rehabilitation, resulting in poor functional prognosis and increased healthcare costs [4,9,10]. Therefore, PSH attacks should be diagnosed early and treated appropriately. β-adrenergic blockers (BBs) suppress blood pressure and heart rate by blocking β-receptors in the sympathetic nervous system and are used to treat hypertension, angina, and tachyarrhythmia. In clinical practice, BBs are available as injectable solutions, oral preparations, and patches; propranolol is available as an oral tablet. The BBs are one of the effective treatments for PSH attacks following severe TBI, and their administration reduces hospital stay and mortality, but the evidence is still limited [11].

Here, we report a case in which propranolol was significantly effective for typical PSH attacks that occurred during the acute phase of diffuse axonal injury.

## Case presentation

**Figure 1:**
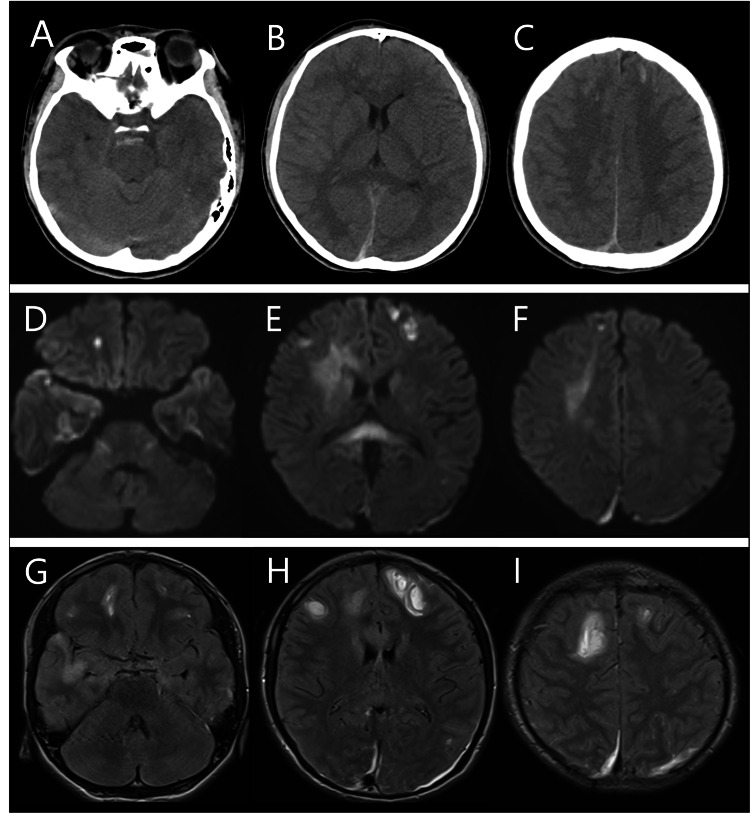
Clinical images. A-C: (Axial views) Computed tomography shows hemorrhages in the bilateral frontal lobes, basilar cisterns, and along the falx cerebri. D-I: (Axial views) Magnetic resonance image performed on the 10th day shows cerebral contusion in bilateral frontal lobes, genu and splenium of the corpus callosum on diffusion-weighted (D-F) and fluid-attenuated inversion recovery images (G-I).

A previously healthy 16-year-old male individual was clashed by a car while riding a bicycle. On arrival at the hospital, his consciousness state was a coma, and his Glasgow Coma Scale score was E1V1M2. The patient had anisocoria (right: 5 mm, left: 3 mm), lower extremities were extended, and there was no response to pain. Vital signs at the emergency room were as follows: temperature 36.5°C, pulse rate 85 beats per minute, and blood pressure 131/80 mmHg. The endotracheal intubation was immediately performed because of presenting with Cheyne-Stokes breathing. A computed tomography scan showed hemorrhages in the bilateral frontal lobes, basilar cisterns, and along the falx cerebri, suggesting elevated intracranial pressure (Figure 1A-1C).

**Figure 2:**
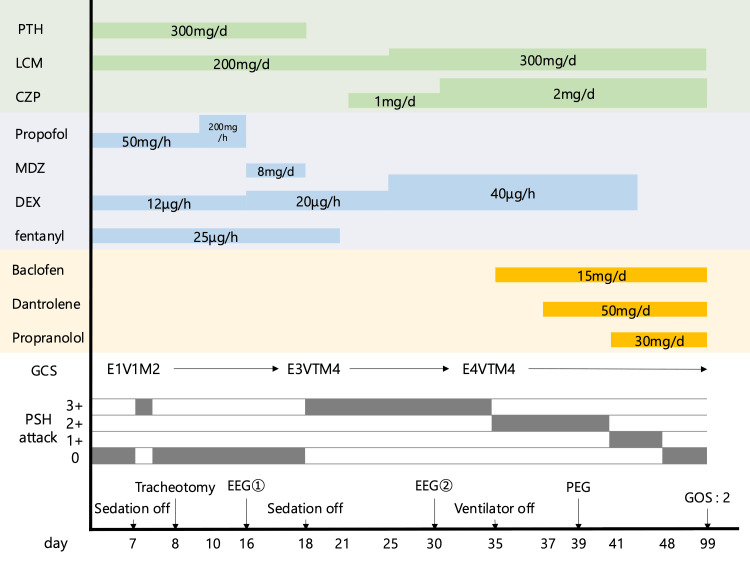
Clinical course. Schematic diagram showing the type of drugs administered, dosage, duration of administration, and clinical course. The severity of PSH attacks is classified according to the number of attacks per day as follows; grade 0: none: grade 1: 1-2, grade 2: 3-5, grade 3: 6 or more. CZP: clonazepam, DEX: dexmedetomidine hydrochloride, EEG: electroencephalogram, GCS: Glasgow Coma Scale, GOS: Glasgow Outcome Scale, LCM: lacosamide, MDZ: midazolam, PEG: percutaneous endoscopic gastrostomy, PSH: paroxysmal sympathetic hyperactivity, PTH: phenytoin.

As there was no intracranial hematoma with mass effect, craniotomy was not performed, and conservative treatment mainly using medication to reduce cerebral edema was initiated. To stabilize the patient's vital signs and prevent increasing intracranial pressure, the patient was given fentanyl as an analgesic and both propofol and dexmedetomidine hydrochloride as sedatives. To suppress acute convulsions caused by cerebral contusion, two antiepileptic drugs, phenytoin 300 mg/day and lacosamide 200 mg/day, were administered from the start of hospitalization. The type of drugs administered, the dosage, duration of administration, and clinical course are shown in a schematic diagram (Figure 2).

**Figure 3:**
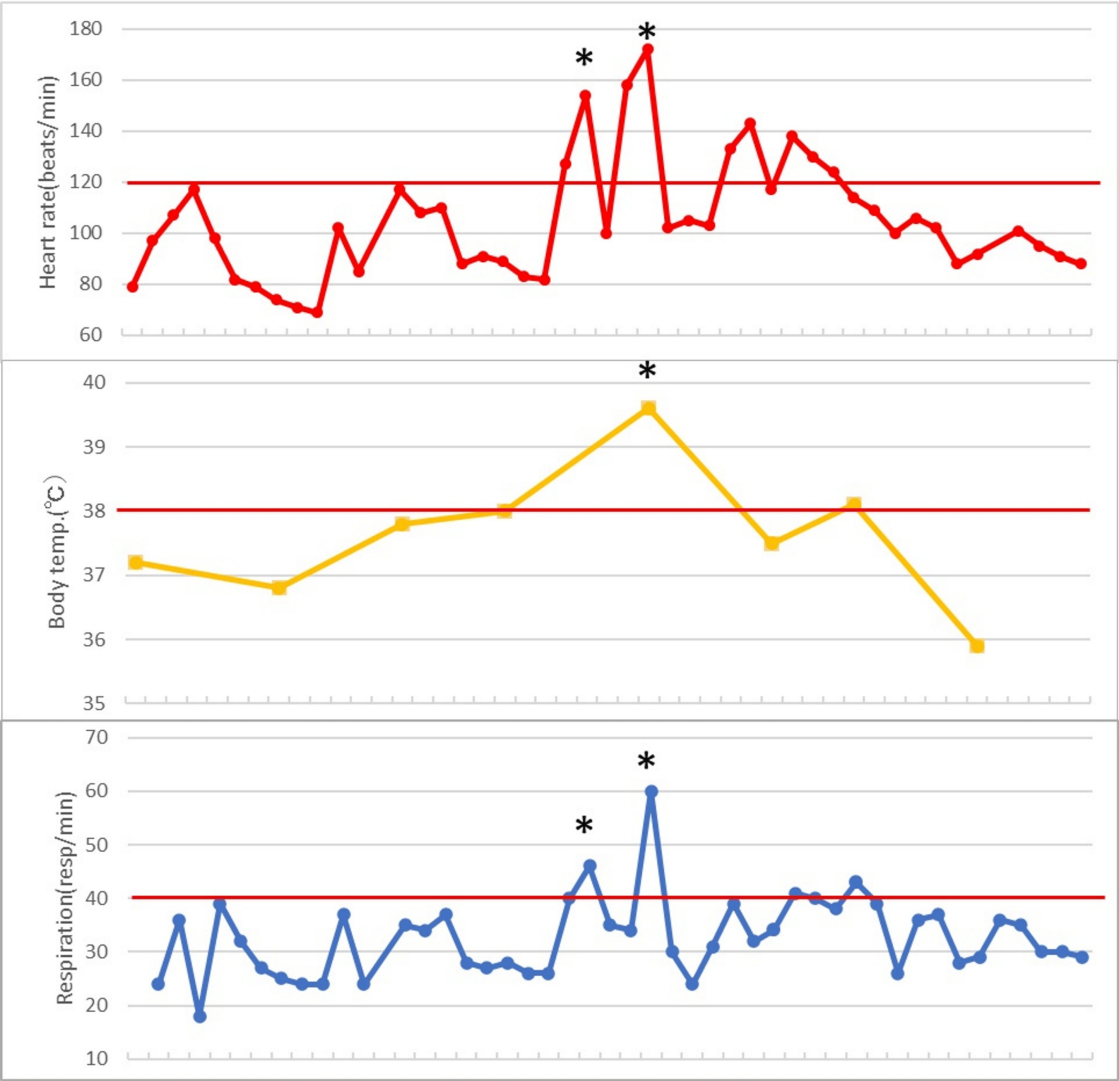
Vital signs. Line graphs showing time courses of the patient's heart rate, body temperature, and respiratory rate during a PSH attack. The thick red horizontal lines indicate the upper normal range for each item. The grid lines on the horizontal axis represent 30-minute intervals. PSH: paroxysmal sympathetic hyperactivity.

On the 7th day, the sedatives were temporarily discontinued, but the patient's limbs were held in an extended position with muscle contractions. At the same time, the body temperature rose to 40.0°C, and the patient also exhibited tachycardia, sweating, and hypertension (Figure 3).

**Figure 4:**
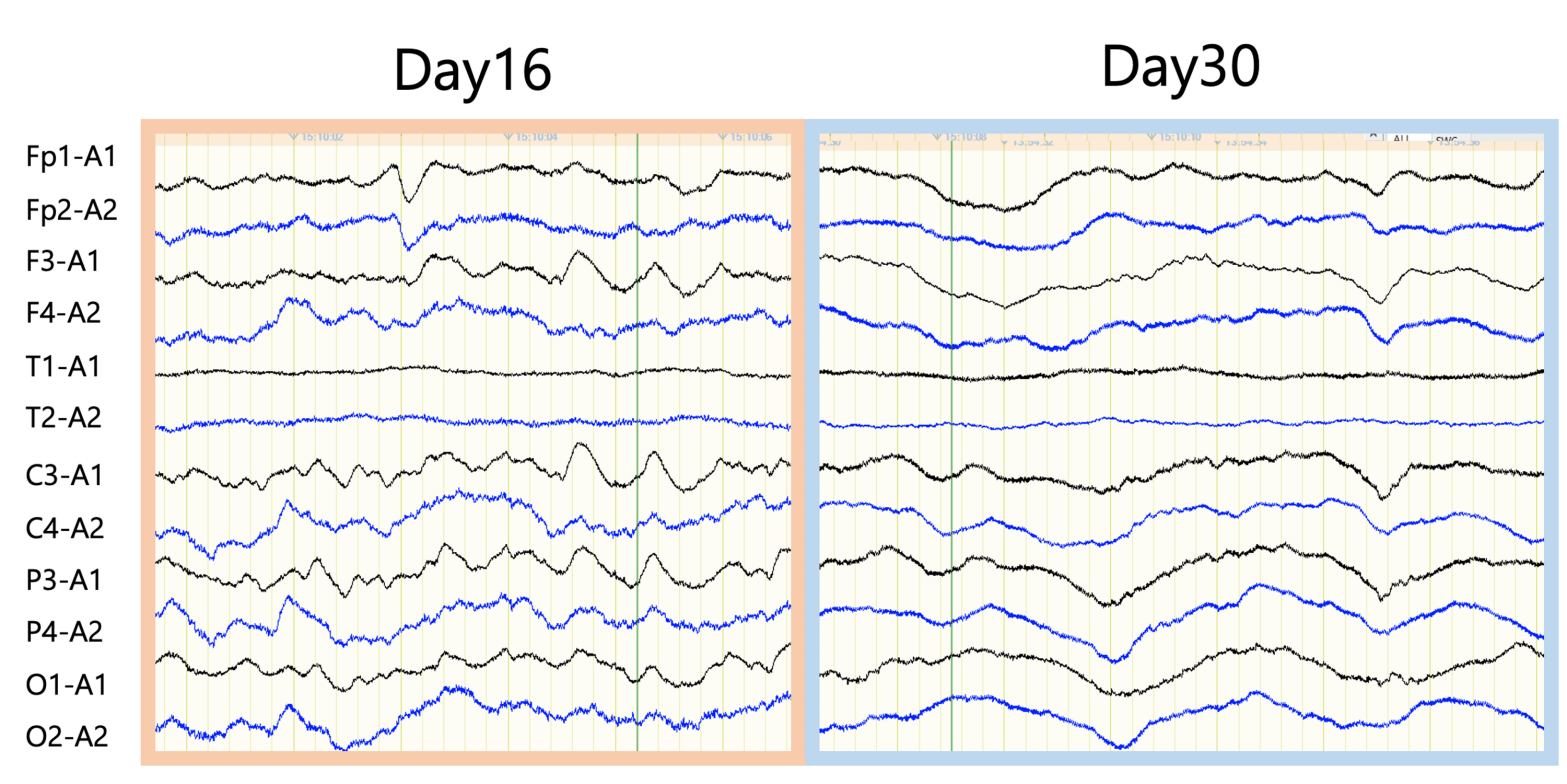
Electroencephalograms. Electroencephalograms recorded with referential derivations performed on the 16th and 30th days show no interictal epileptic discharges (left: 16th day, right: 30th day).

These attacks lasted approximately 30 minutes and occurred multiple times a day. These attacks could not be controlled with administration of fast-acting intravenous antiepileptic drugs but resolved after re-administration of sedatives. Blood test results showed a white blood cell count of 8,000/μl and C-reactive protein of 3.40 mg/dl, and various cultures (blood, sputum, and urine) were negative, ruling out bacterial infection. After re-administration of sedatives, magnetic resonance imaging and electroencephalogram (EEG) were performed. Magnetic resonance imaging showed findings of cerebral contusion in bilateral frontal lobes, genu and splenium of the corpus callosum, leading to a diagnosis of diffuse axonal injury (Figure 1D-1I). On the 8th day, a tracheotomy was performed. EEGs were performed twice on the 16th and 30th days, but no epileptic discharges were detected at either time (Figure 4).

On the 18th day, sedatives were discontinued again, but similar symptoms recurred and sedatives could not be discontinued. On the 35th day, the patient was able to be weaned from mechanical ventilatory support. On the 39th day, percutaneous endoscopic gastrostomy was performed. The muscle stiffness was slightly alleviated by adding baclofen and dantrolene, but similar attacks occurred when sedation was discontinued. As a result, the patient was unable to be weaned off intensive care unit management and continuous administration of dexmedetomidine hydrochloride. Based on the clinical course and various examination results, these symptoms were judged to be PSH attacks, and administration of propranolol 30 mg/day was started on the 41st day. The severity of PSH attacks was classified according to the number of attacks per day as follows: grade 0: none, grade 1: 1-2, grade 2: 3-5, grade 3: 6 or more. After starting propranolol administration, PSH attacks decreased and disappeared completely, and finally dexmedetomidine hydrochloride could be discontinued (Figure 2). On the 99th day, the patient was transferred to a rehabilitation hospital. Three months later, he could ride in a wheelchair but he was unable to communicate nor take oral nutrition, and required full assistance.

## Discussion

PSH is a syndrome of simultaneous, paroxysmal, transient increases in sympathetic nervous activity and motor (posturing) activity that occurs in some survivors of severe acquired brain injury [1]. According to a review, the causes of brain injury leading to PSH were TBI in 79.4% of cases, hypoxia in 9.7% of cases, and cerebrovascular disease in 5.4% of cases [3]. Meanwhile, the incidence of PSH in intensive care unit TBI patients has been estimated to range from 7.7% to 33% [9,12]. Patients with PSH generally have a poor prognosis, with 18% death and 30% remaining in a vegetative state [3]. 

There are several theories on the pathophysiological mechanism of PSH, including disconnection theory, neuroendocrine theory, and neutrophil extracellular trap theory, but excitatory/inhibitory ratio theory is the most accepted [6,7,13]. The excitatory/inhibitory ratio theory is based on the idea that a balance between cerebral inhibition and spinal input produces appropriate sympathetic and motor output for stimuli. Spinal levels provide upward feedback of sensory and perceptual stimuli and output sympathetic and motor efferent. Cerebral levels such as the cerebral cortex, thalamus, and hypothalamus inhibit these spinal reflexes. Severe brain damage impairs cerebral inhibition, causing excessive spinal reflexes even in response to non-noxious stimuli such as aspiration sputum, resulting in PSH attacks due to excessive sympathetic nerve output [13].

To resolve the confusion regarding the terminology and disease concept of this syndrome, a definition, diagnostic criteria, and PSH assessment scale were established in 2014 [1]. In this case, the total score was 27 points and classified as probable PSH. The assessment scale consists of two subscales: the clinical feature scale and the diagnosis likelihood tool. The clinical features scale includes the following six items: heart rate, respiratory rate, systolic blood pressure, temperature, sweating, and posturing. Each item is scored as 0: within normal range, 1: mild abnormality, 2: moderate abnormality, and 3: severe abnormality, with a total range of 0 to 18. The diagnosis likelihood tool consists of following 11 items and is scored on ranging from 0 to 11. Each item is scored 1 if present and 0 if absent: clinical features occur simultaneously, episodes are paroxysmal, sympathetic over-reactivity to normally non-painful stimuli, features persist over three consecutive days, features persist over two weeks post-brain injury, features persist despite treatment of alternative differential diagnoses, medication administered to decrease sympathetic features, more than twoi episodes daily, absence of parasympathetic features during episodes, absence of other presumed cause of features, antecedent acquired brain injury. The sum of both subscales is calculated to determine the likelihood of PSH as follows: <8: unlikely; 8-16: possible; ≥ 17: probable.

Early detection and systematic management may lead to good clinical outcomes [14]. However, early diagnosis is difficult because patients presenting with PSH are often critically severe conditions and sedated during the acute phase, and PSH is essentially a diagnosis of exclusion. Bacterial sepsis should be ruled out for high fever, tachycardia, and elevated blood pressure of PSH attacks, and intracranial abnormalities such as secondary epilepsy, hydrocephalus, and increased intracranial pressure should be ruled out for tonic-seizure-like or dystonia-like symptoms. There is no established treatment for PSH, and treatment has been tailored to the patient’s clinical symptoms and comorbidities. Medications used for PSH include skeletal muscle relaxants and γ-aminobutyric acid receptor modulators to reduce spasticity, and α2 agonists and long-acting benzodiazepines for sedation [2,13]. In this case, the PSH attacks could not be suppressed by administration of a skeletal muscle relaxant (dantrolene) and γ-aminobutyric acid receptor modulator (baclofen) via a gastrostomy tube, but the attacks were suppressed by adding continuous intravenous administration of an α2 agonist (dexmedetomidine hydrochloride). However, discontinuation of the sedative infusion resulted in the recurrence of PSH attacks, preventing rehabilitation interventions to restore function.

The surge of catecholamines caused by severe TBI leads to hyperthermia, tachycardia, tachypnea, diaphoresis, and hypertension, which can lead to secondary brain damage [15]. The effectiveness of BBs administration for treating symptoms of catecholamine surge has been shown, and propranolol in particular may be more beneficial for TBI patients than other BBs [11,16,17]. First, propranolol is lipophilic, allowing it to penetrate the central nervous system well [16]. Second, propranolol increases cerebral perfusion and improves oxygen supply in mouse models [17]. Finally, propranolol contributes to reducing hospital stay and mortality in patients with severe TBI [11]. In clinical practice, combinations of medications are commonly used to treat PSH [18]. In this case, PSH attacks were suppressed by combining the administration of dantrolene, baclofen, and propranolol via a gastrostomy tube, enabling the patient to be weaned off a continuous intravenous infusion of sedatives and begin rehabilitation. The administration of baclofen via a gastrostomy tube must be in high doses to be effective because baclofen has poor blood-brain barrier permeability [19]. In contrast, intrathecal baclofen therapy is a surgical treatment that can reduce the frequency of PSH attacks with very low doses of baclofen and is an effective treatment option for refractory chronic PSH [20].

In this case, PSH attacks, with symptoms such as hyperthermia, tachycardia, sweating, hypertension, and extensor posturing, were repeated during the acute phase of severe TBI. Sepsis and epilepsy were ruled out by various culture tests and EEGs, but continuous intravenous infusion of sedatives could not be discontinued due to the suppression of PSH attacks. The addition of propranolol via a gastrostomy tube dramatically resolved the PSH attacks, and the patient was transferred to a rehabilitation hospital.

## Conclusions

Paroxysmal sympathetic hyperactivity occurs primarily after severe traumatic brain injury and is characterized by abnormal paroxysmal surges in sympathetic nervous system activity. A 16-year-old male individual with severe diffuse axonal injury suffered from sudden increases in heart rate, respiratory rate, blood pressure, body temperature, diaphoresis, and extended posturing. Because these symptoms appear suddenly, it is called a "PSH attack." His PSH attacks recurred over a month and intravenous dexmedetomidine was required. We administrated propranolol via a gastrostomy tube and PSH attacks decreased and disappeared completely, and dexmedetomidine could be discontinued. Early detection and systematic management of PSH may lead to good clinical outcomes. Propranolol is one of the effective treatments for paroxysmal sympathetic hyperactivity.
